# A Neural Network with Convolutional Module and Residual Structure for Radar Target Recognition Based on High-Resolution Range Profile

**DOI:** 10.3390/s20030586

**Published:** 2020-01-21

**Authors:** Zhequan Fu, Shangsheng Li, Xiangping Li, Bo Dan, Xukun Wang

**Affiliations:** Coast Defense College, Naval Aviation University, Yantai 264001, China; liss1965@163.com (S.L.); lixiangping401@126.com (X.L.); lovelin19841204@163.com (B.D.); wang17616244926@163.com (X.W.)

**Keywords:** neural network, target recognition, HRRP, residual structure, loss function

## Abstract

In the conventional neural network, deep depth is required to achieve high accuracy of recognition. Additionally, the problem of saturation may be caused, wherein the recognition accuracy is down-regulated with the increase in the number of network layers. To tackle the mentioned problem, a neural network model is proposed incorporating a micro convolutional module and residual structure. Such a model exhibits few hyper-parameters, and can extended flexibly. In the meantime, to further enhance the separability of features, a novel loss function is proposed, integrating boundary constraints and center clustering. According to the experimental results with a simulated dataset of HRRP signals obtained from thirteen 3D CAD object models, the presented model is capable of achieving higher recognition accuracy and robustness than other common network structures.

## 1. Introduction

The high-resolution range profile (HRRP) of a target refers to the projection of the target scattering center following the radar line of sight, covering numerous target characteristics (e.g., size and structure). HRRP can be acquired, processed and stored easily; it has a simplified computation and robust real-time performance. For this reason, it has constantly been a critical data source for target recognition. Researchers are able to harvest separable features from HRRP to classify and identify a range of targets. Previous HRRP-based radar target classification and recognition placed primary emphasis on feature extraction on the basis of researchers’ prior knowledge and experience, as well as optimization and fusion of classification algorithms. Common features consist of time domain characteristics [1,2] (as manifested by original image, central moment, structure contour, strong scattering points, etc.), while power spectrum, polarization ratio, polarization matrix and other frequency domain [3,4] and polarization domain [5,6] characteristics are also covered.

Fueled by advances in computer technology, and in accordance with deep learning theory, deep learning has become a hotspot in research in various fields [7,8,9,10]. It has been extensively employed in radar target detection, recognition, and classification. At the same time, HRRP and CNN also have significant applications in the field of unmanned aerial vehicles and unmanned surface vehicles [11,12]. Deep learning-based object recognition refers to feature extraction using a neural network. HRRP-based radar recognition can also be achieved using a deep learning algorithm. This field has aroused a great deal of attention from researchers, and considerable new achievements have been made, which will be presented below. There are many methods for enhancing the recognition accuracy of neural networks, such as improving the structure of the neural network, optimizing the loss function, and increasing the training data. To be specific, the neural network structure for HRRP target recognition consists of an autoencoder (AE) and a convolutional neural network (CNN).

CNN refers to a critical deep learning structure, capable of automatically extracting the effective separable characteristics of HRRP and addressing the susceptibility to amplitude, translation and orientation that results from the structural similarity between different ships. Compared with the conventional classification algorithm, it exhibits a better recognition effect. In [13], CNN was employed to identify aircraft based on HRRP, and an analysis was conducted on the effects of activation function, convolution kernel size, learning rate and weight decay coefficient on recognition accuracy. It exhibited higher recognition accuracy than those of back propagation (BP) neural network, support vector machine (SVM), and K-nearest neighbor (KNN). Their dataset originated from actual measurements of four aircraft scale models. In [14], CNN was also adopted to achieve target recognition, with the dataset being derived from the simulation calculation of 10 ship targets. In [15], when CNN was employed for target recognition, white noise was added to expand the dataset. Moreover, the recognition results of different radars were fused, and the threshold was set to determine whether the target was known or unknown. Furthermore, the effect of radar numbers and SNR on recognition accuracy was analyzed. In [16], an algorithm that integrates HRRP with polarization information was proposed. With the use of the polarization matrix, Pauli decomposition and Freeman decomposition, 12 eigenvectors were achieved to form the dataset. According to the simulation result, the recognition accuracy based on fully polarized datasets was 5 percentage higher than that of single polarized datasets. Similar work was also conducted in [17], wherein the dataset originated from full-polarization measurements of the model using 77 GHz electromagnetic waves in a microwave anechoic chamber. In [18], two CNN models with various structures were built, and the difference in recognition effect was studied.

AE refers to a type of data compression algorithm, capable of reproducing the input signal to the greatest extent by harvesting the crucial features of the input data. The vital features extracted can be exploited to identify the target. In [19], a deep network, termed a sparse convolution autoencoder (S_1_C_1_AE), was presented to deal with HRRP target recognition. The model was employed to identify 3 vehicle models. While data were being preprocessed, amplitude normalization and centroid alignment were performed. As compared with other models, the recognition accuracy was enhanced noticeably. Comparison models covered linear discriminate analysis (LDA), principal component analysis (PCA), linear support vector machine (LSVM), denoise sparse autoencoder (D_1_S_1_AE), and deep belief network (DBN). In [20], a stacked corrective autoencoder (S_2_C_2_AE) was built, and the data preprocessing was the same as the process in [19]. The correction was achieved by averaging HRRP for respective frame. The model exhibits better generalization performance. To form a loss function based on the Mahalanobis distance, the covariance matrix of each HRRP was employed. Experimental results suggested that the deeper layers of the model, the better the recognition effect could be. In [21], a HRRP recognition model was proposed, combining S_1_C_1_AE and multiple classifiers. First, the S_1_C_1_AE was employed to extract features, and subsequently the random forest (RF), naive Bayes (NB) and minimum classifier were fused for features classification. According to experimental results, the model exhibited good noise robustness. In [22,23,24], the recognition method of fusion neural networks and classifiers was also studied.

There are no publicly available datasets for deep learning-based HRRP target recognition. Most of the datasets adopted by the various researchers of HRRP target recognition are derived from measurements in a microwave anechoic chamber, as well as from their simulation calculation. Nevertheless, different studies have suggested that deep learning-based HRRP target recognition exhibits higher accuracy than conventional classification methods. At present, the enhancement of HRRP target recognition accuracy based on neural networks is primarily focused on the enhancement and fusion of the mentioned structures. In contrast, only rare studies have sought to enhance the accuracy of HRRP target recognition by optimizing the loss function. Most studies that optimize the loss function to enhance the recognition effect are aiming towards face recognition, and many of them can be applied to HRRP target recognition.

Designing a good neural network structure is one of the most efficient and challenging approaches to enhancing classification performance. Under the premise of sufficient datasets, the learning ability of the model can be enhanced by up-regulating the depth and width of the neural network. AlexNet [25] and VGG [26] have both demonstrated that model recognition accuracy displays a positive correlation with the network depth in a certain range. Nevertheless, with the increase in network depth, gradient explosion, disappearance, and saturation of network recognition accuracy may take place in the back propagation of CNN in the training process. By introducing a residual learning framework, Kaiming He and Xiangyu Zhang [27] addressed the degradation problem. Accordingly, the problem whereby the accuracy reaches saturation and subsequently degrades rapidly with the rise in the network depth was avoided. However, to enhance the recognition effect, the residual learning framework requires further increases in the network depth.

In this study, an efficient and extensible convolutional module is presented by optimizing the residual learning framework. The convolutional module contains left and right branches. Among them, based on the left branch structure of convolutional module, the effect of network deepening and widening can be simulated. The skip structure of the right branch is capable of transferring features and gradients more effectively. The convolutional module is capable of achieving the recognition effect of a deep network with fewer network parameters. Additionally, a novel loss function is proposed to enhance the recognition accuracy by combining central clustering and additive margin strategy. The features extracted by the novel loss function are characterized by larger inter-class variations, smaller intra-class variations, and stronger separability. In the meantime, by combining convolutional module that exhibits the same topology, the presented model can be extended to adapt to various difficulty classification tasks. According to the experimental results with a simulated dataset of HRRP, the presented model is capable of achieving higher recognition accuracy than the conventional algorithm. The rest of this paper is organized as follows. Section 2 presents the composition and structure of one-dimensional convolutional network. The design of convolutional module and loss function are elucidated in Section 3. The experimental effect of the model is demonstrated in Section 4 from different aspects. Lastly, the concluded remarks are drawn in Section 5.

## 2. One-Dimensional Convolutional Neural Network

**Figure 1:**
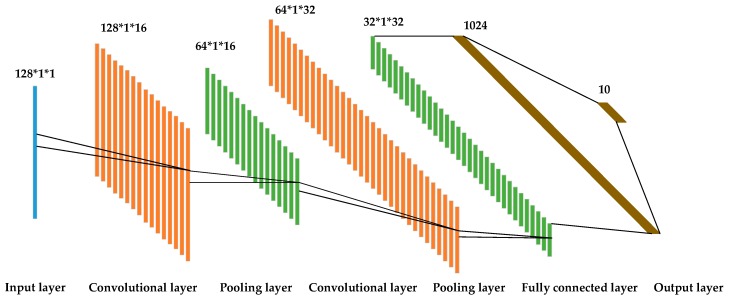
Schematic diagram of the CNN structure for HRRP. It shows CNN’s classification process of 10 types of targets for 128-length HRRP data.

CNN refers to a type of feedforward neural network that covers convolution calculation. For its translation invariance in the calculation process, it is capable of avoiding complex preprocessing (e.g., HRRP data alignment) and exhibits higher robustness. The model employed in this study complies with the convolutional neural network. First, the basic structure of CNN is introduced, which covers five parts, namely, input layer, convolutional layer, pooling layer, fully connected layer, and output layer. The CNN structure for HRRP is illustrated in Figure 1.

The input layer acts as the start of the neural network, generally requiring simple preprocessing of data to make the data have the identical dimension and satisfy the same distribution characteristics. Preprocessing is capable of down-regulating the effect of amplitude perturbation on the extraction characteristics of different HRRP data and enhancing the robustness of the model. It is also convenient to find the minimum value more directly in the iterative process of the gradient descent method, so the model can converge faster. It can be performed in the two steps below:

1. Normalize the amplitude of HRRP. The data after amplitude normalization of the *n*th HRRP is expressed as ${x^{\prime }}_{n}=x_{n}/\max\left(\left|x_{n}\right|\right)$, where $\max\left(\left|x_{n}\right|\right)$ denotes the maximum absolute value of all elements in HRRP.
2. Subtract the mean value of the normalized HRRP data from the respective element.

The major function of the convolutional layer is to extract the features of the input data. In Figure 1, the first convolutional layer covers 16 convolution kernels, and the second convolutional layer consists of 32 convolution kernels. Each convolution kernel element is composed of weight coefficient and bias. In deep learning, the weight coefficient initialization method of the neural network plays an important role in the convergence speed and performance of the model. Common weight coefficient initialization methods include random initialization, Xavier initialization [28], and He initialization [29]. Random initialization may cause gradient disappearance when the neural network layers are deep. To solve this problem, the Xavier initialization method was proposed. When used in conjunction with the Tanh activation function, the Xavier initialization method makes the output value of the activation function of the network layer obey the Gaussian distribution. The generation of gradient disappearance is avoided. However, when used with the Relu activation function, the problem of gradient disappearance still exists. The He initialization method proposed in [29] solves the problem of gradient disappearance when the Relu activation function is used in combination with it. The convolution kernel calculates the input data by convolution, adds the bias, and then activates it by means of the activation function. The output of the convolutional layer is the extracted feature. The calculation process can be written as:

(1) $$x_{j}^{l}=f\left(\sum_{i\in M_{j}}x_{i}^{l-1}*k_{ij}^{l}+b_{j}^{l}\right),$$

where $x_{j}^{l}$ denotes the output of the jth channel, belonging to the lth convolutional layer. f(⋅) refers to the activation function, employing the Relu function. $k_{ij}^{l}$ is the convolution kernel vector of the jth channel of the convolutional layer l that corresponds to the ith input vector. $b_{j}^{l}$ is the bias of the jth channel of the convolutional layer l, * represents the convolution operation. The parameters of the convolutional layer consist of convolution kernel size, step size, filling category, as well as activation function. The common activation functions cover the Sigmoid function, the Relu function, etc. For various parameters, the convolutional layer exhibits different characteristics.

The function of the pooling layer aims to select the features extracted by the convolutional layer and down-regulate the dimension by down-sampling. Max-pooling, mean-pooling and mix-pooling are the common pooling layers.

On the whole, the fully connected layer is placed on the back side of the neural network. The major function is to arrange the features extracted from the previous layer to yield the one-dimensional vector. The whole CNN outputs target-related outcomes through the output layer classifier. The common classifiers are softmax and SVM. In the task of target recognition, the output of CNN can cover the category, size and central coordinates of the target. The learning process of CNN usually updates parameters iteratively by back propagation, and stable identification results are obtained by minimizing the error calculated by the loss function.

## 3. Model Analysis and Design

### 3.1. Design of Convolutional Module

**Figure 2:**
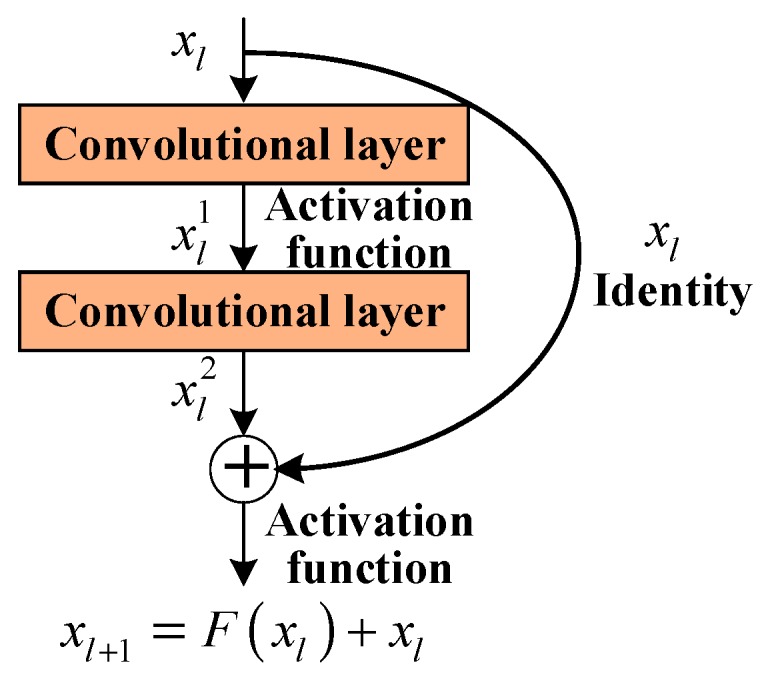
Schematic diagram of residual block.

The depth of neural networks is critical. The deep convolutional neural network is capable of extracting and fusing features of different levels for end-to-end target recognition. Nevertheless, the deepening of network layers will cause saturated recognition accuracy. To address this problem, residual structure is introduced, as illustrated in Figure 2.

The residual block in the residual structure consists of convolutional layers, and the number of convolutional layers in Figure 2 is 2. The residual structure outputs the sum of the input feature, and the output of the last convolutional layer is expressed by

(2) $$x_{l+1}=F\left(x_{l}\right)+x_{l},$$

where $x_{l}$ and $x_{l+1}$ represent the input and output feature vector of the residual block, respectively. $F\left(x_{l}\right)$ denotes the mapping of residual blocks.

**Figure 3:**
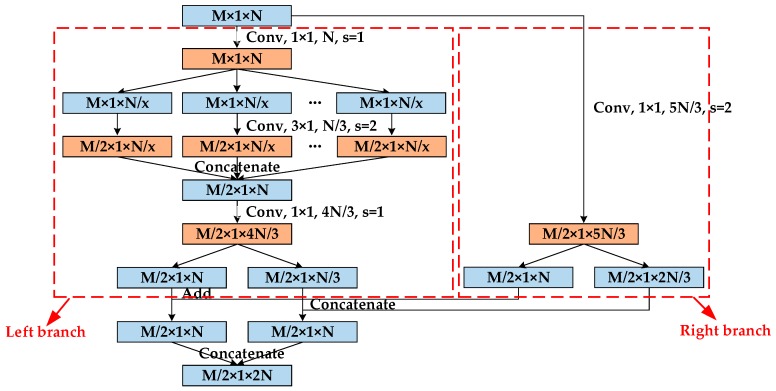
Structure of the convolutional module. M × 1 × N represents one-dimensional data with input characteristics of M × 1, N feature layers, s is the moving step size of the convolution kernel, and the unmarked step size is 1 by default.

Research results reveal that the saturated recognition accuracy of deep network can be effectively addressed by replacing the required fitting mapping $F\left(x_{l}\right)+x_{l}$ with the fitting mapping $F\left(x_{l}\right)$ [27]. In particular, if the network has extracted the optimal features required for classification, the residual structure should only carry out identity mapping of skip connections to ensure the maximal recognition accuracy. For neural networks, zero residual block is more efficient than the use of multilayer neural networks to fit identity mapping. Figure 3 presents the structure of the convolutional module promoted in this study based on the residual structure, where conv denotes the convolutional layer.

The convolutional module proposed in this article is set up as a highly modular network structure that exhibits high expansibility. The features extracted by the upper layer network act as the input of this layer, and the input will pass through two branches, as shown in Figure 3. In the left branch, the convolution kernel of 1 × 1 is adopted to fuse the features between layers first. Subsequently, the fused features are split into x branches according to the number of layers. Each branch contains 3 layers of features, and all branches adopt a convolution kernel of 3 × 1 to extract features; the step size is 2. Since the step size of the convolution kernel is 2, the number of layers of the output feature remains unchanged, and the dimension is halved. Next, the features of all branches are concatenated. Moreover, the size of x can be ascertained according to the complexity of the classification tasks. The larger x is, the easier it is to extract stronger separable features, and the better the recognition effect is in a more complex classification task. Such structure is similar to Inception [30]. Nevertheless, the size and number of the convolution kernel for each branch in Inception are customized step by step. In the convolutional module proposed in this article, a small-scale convolution kernel of 3 × 1 is uniformly chosen to simplify the structure design and ensure the recognition effect in the meantime. After concatenation, the features are fused again with the convolution kernel of 1 × 1, and the number of feature layers is then up-regulated. In the left branch in Figure 3, the number of feature layers increases from N to 4N/3. Then, according to the number of layers, the features are split into two parts to prepare for the subsequent fusion of the features of the two branches, where the number of layers of features for add is N, and the number of layers of features for concatenate is N/3, as shown in Figure 3. The right branch directly uses the convolution kernel of 1 × 1 to fuse the input features and rises the number of feature layers. In the meantime, the features are also separated into two parts according to the number of layers. The number of layers of features for add is N, and the number of layers of features for concatenate is 2N/3. Lastly, the corresponding features in the left and right branches are added or concatenated, as illustrated in Figure 3.

Compared with the input of the convolutional module, the dimension of the output features is halved, and the number of layers is doubled. The right branch exerts similar effects as the residual network, making the transfer of features and gradients more efficient. Because of the right branch, each layer of convolutional module is capable of acquiring information from the loss function and the original input, and the exploitation of shallow features is facilitated. Then, the problem that the recognition accuracy decreases with the rise in the number of network layers is avoided.

### 3.2. Design of Loss Function

The loss function is adopted to identify the difference between the predicted value and the real value. Softmax loss commonly acts as the loss function for multi-classification convolutional neural network. However, from the clustering perspective, the feature extracted from softmax loss will display larger intra-class variations than inter-class variations. In the meantime, the features extracted by softmax loss are not discriminative enough, since they still display significant intra-class variations. Under too many target types, the features will overlap, which is not conducive to object classification. To solve this problem, numerous solutions have been proposed in face recognition [31,32,33,34,35]. They primarily focus on promoting inter-class variations and lowering intra-class variations.

For softmax loss, features can be brought closer by enhancing the boundary constraints between various targets. It also promotes the inter-class variations of targets. The formulation of the original softmax loss is defined as:

(3) $$\begin{array}{cc} L_{S} & =-\frac{1}{m}\sum_{i=1}^{m}\log\frac{e^{W_{y_{i}}^{T}x_{i}}}{\sum_{j=1}^{n}e^{W_{j}^{T}x_{j}}}\\ & =-\frac{1}{m}\sum_{i=1}^{m}\log\frac{e^{\left\|W_{y_{i}}\right\|\left\|x_{i}\right\|\cos(\theta_{y_{i}})}}{\sum_{j=1}^{n}e^{\left\|W_{j}\right\|\left\|x_{i}\right\|\cos(\theta_{j})}} \end{array}$$

where x represents the input of the last fully connected layer. $x_{i}\in {\mathbb{R}}^{d}$ denotes the ith deep feature, belonging to the $y_{i}\mathrm{th}$ class. d indicates the feature dimension. $W_{j}\in {\mathbb{R}}^{d}$ refers to the jth column of the weights $W\in {\mathbb{R}}^{d\times n}$ in the last fully connected layer. $W_{y_{i}}^{T}x_{i}$ denotes the target logit of the ith sample. m and n represent the size of mini-batch and the number of class, respectively. 

The design of the loss function proposed refers to the additive margin softmax loss (AM-softmax), which is used in face recognition [35]. In the meantime, considering the constraint of intra-class variations of features, a loss function named margin center (Referred to MC), integrating additive margin and center constraint, is proposed. The loss function uses the additive margin to increase the inter-class variations of features; the center constraint is also employed to reduce the intra-class variations of features. As a result, the inter-class variations of features are larger, the intra-class variations are smaller, and the separability of features is enhanced. The formulation of the loss function proposed in this study is given by

(4) $$\begin{array}{cc} L_{AMSC} & =L_{AMS}+\lambda L_{C}\\ & =-\frac{1}{m}\sum_{i=1}^{m}\log\frac{e^{s\cdot (W_{y_{i}}^{T}x_{i}-\mu )}}{e^{s\cdot (W_{y_{i}}^{T}x_{i}-\mu )}+\sum_{j=1,j\neq y_{i}}^{n}e^{sW_{j}^{T}x_{i}}}+\frac{\lambda }{2}\sum_{i=1}^{m}{\left\|x_{i}-c_{y_{i}}\right\|}_{2}^{2}\\ & =-\frac{1}{m}\sum_{i=1}^{m}\log\frac{e^{s\cdot (\cos\theta_{y_{i}}-\mu )}}{e^{s\cdot (\cos\theta_{y_{i}}-\mu )}+\sum_{j=1,j\neq y_{i}}^{n}e^{s\cdot \cos\theta_{j}}}+\frac{\lambda }{2}\sum_{i=1}^{m}{\left\|x_{i}-c_{y_{i}}\right\|}_{2}^{2} \end{array}$$

where the hyper-parameter s is adopted to scale the cosine values, and cosine values represent the similarity between the features. μ is applied for the control of the distance between the edges of the feature. $c_{y_{i}}\in {\mathbb{R}}^{d}$ denotes the $y_{i}\mathrm{th}$ class center of features, and $c_{y_{i}}$ can constantly update with the variation of the features of each batch. $L_{AMS}$ proposes a specific ψ(θ)=cosθ−μ to introduce the additive margin property and enhances the recognition effect by promoting the inter-class variations of features. $L_{C}$ constructs a class center for the features of each class of target and punishes the features far away from the class center. Accordingly, the intra-class variations of features becomes more compact, the intra-class variations are lowered, and the inter-class variations are promoted. The gradients of $L_{C}$ with respect to $x_{i}$ and update equation of $c_{y_{i}}$ are computed as:

(5) $$\frac{\partial L_{C}}{\partial x_{i}}=x_{i}-c_{y_{i}},$$

(6) $$\Delta c_{j}=\frac{\sum_{i=1}^{m}\delta (y_{i}=j)\cdot (c_{j}-x_{i})}{1+\sum_{i=1}^{m}\delta (y_{i}=j)},$$

where δ(⋅)=1 if the identification is correct; otherwise, δ(⋅)=0. Under the constraint of joint loss function $L_{AMSC}$, the learning details in network can be summarized in the following (Algorithm 1):
**Algorithm 1. The neural network algorithm with convolution module and residual structure****Input:** Training samples $\left\{x_{i}\right\}$. Initialized parameters $\theta_{c}$ in convolution kernel. Weight matrix W. The jth class center $c_{j}$ of features. Hyper-parameter s, μ in $L_{AMS}$. Learning rate α for feature center in $L_{C}$. Weight λ and learning rate lr in network. The number of iteration t←0.**Output:** The parameters $\theta_{c}$.Step 1: **while** not converge **do**Step 2: t←t+1.Step 3: compute the joint loss by $L_{AMSC}^{t}=L_{AMS}^{t}+\lambda L_{C}^{t}$.Step 4: compute the backpropagation error $\frac{\partial L_{AMSC}^{t}}{\partial x_{i}^{t}}$ for each i by $\frac{\partial L_{AMSC}^{t}}{\partial x_{i}^{t}}=\frac{\partial L_{AMS}^{t}}{\partial x_{i}^{t}}+\lambda \cdot \frac{\partial L_{C}^{t}}{\partial x_{i}^{t}}$.Step 5: update the parameters W by $W^{t+1}=W^{t}-lr\cdot \frac{\partial L_{AMSC}^{t}}{\partial W^{t}}=W^{t}-lr\cdot \frac{\partial L_{AMS}^{t}}{\partial W^{t}}$.Step 6: update the parameters $c_{j}$ by $c_{j}^{t+1}=c_{j}^{t}-\alpha \cdot \Delta c_{j}^{t}$.Step 7: update the parameters $\theta_{c}$ by $\theta_{c}^{t+1}=\theta_{c}^{t}-lr\sum_{i}^{m}\frac{\partial L_{AMSC}^{t}}{\partial x_{i}^{t}}\cdot \frac{\partial x_{i}^{t}}{\partial \theta_{c}^{t}}$.Step 8: **end while**

### 3.3. Design of Model Structure

**Figure 4:**
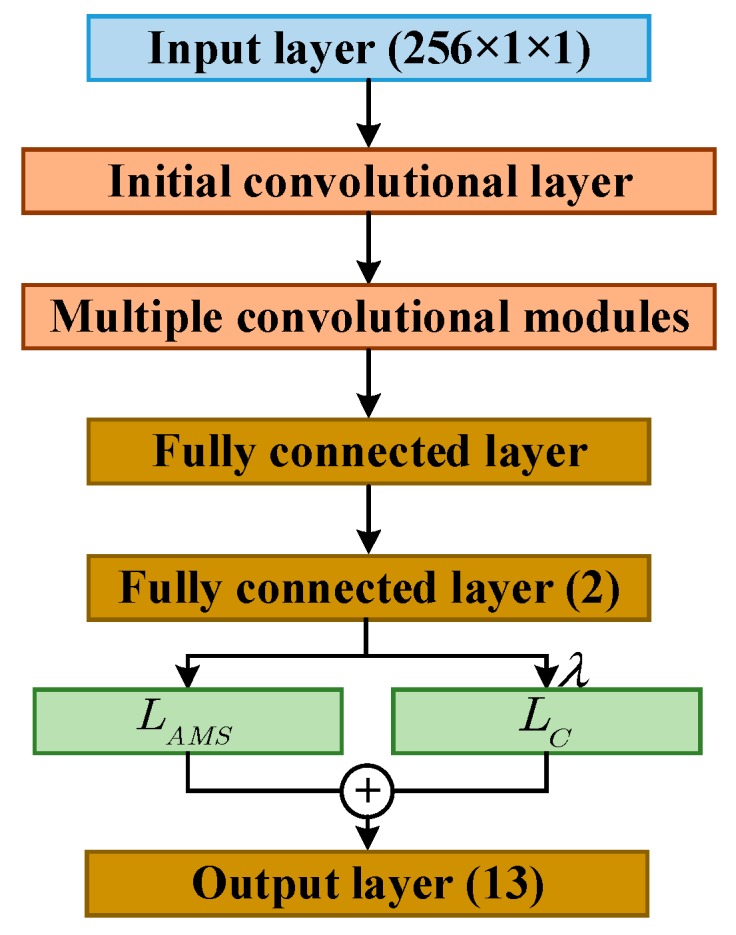
The structure of the presented model.

The block diagram of the presented model in this study is shown in Figure 4, which primarily includes an initial convolutional layer, several convolutional modules with the same topology connected sequentially, and the last two fully connected layers. The dimension of the latter fully connected layer is 2, which is conducive to visualizing the features extracted by the model and analyze the clustering effect of the features.

In Figure 4, the numbers in brackets represent the data dimension after the data passes through this layer, consistent with Figure 3. The output data dimensions of each convolutional module and the first fully connected layer are determined according to the number of convolutional modules. Lastly, the result of the output layer is one-dimensional data that represents the target types. The number of target types in this study is 13. In the presented model, a one-dimensional convolution kernel with a scale of 7 × 1 is taken for the initial convolutional layer. The selection of convolution kernel with relatively large scale in the first layer of the network is conducive to the extraction of the features (e.g., contour and texture in the HRRP). After each convolution operation in this model, batch normalization and Relu activation are performed on the extracted features. Since the Relu activation function is used, the He initialization method is chosen for all weight initialization of the model proposed.

## 4. Experimental Simulation and Analysis

### 4.1. Data Set Construction

**Figure 5:**
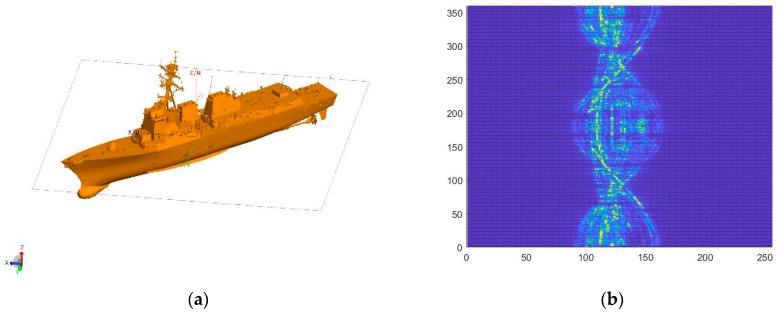
Ship model and the graph of HRRP after amplitude normalization. (**a**) Ship model; (**b**) The graph of HRRP after amplitude normalization.

**Figure 6:**
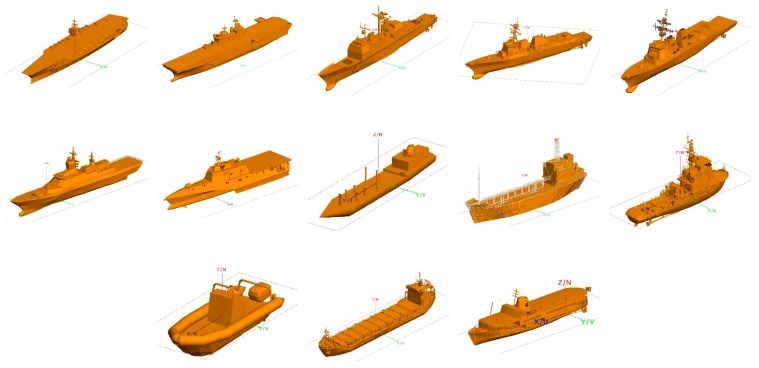
Models of all the ship targets.

On the whole, there are two ways to obtain the target echo signal, namely the measured method and the theoretical calculation method. Since most ship targets are non-cooperative targets, it is very difficult to obtain the HRRP from field measurement. In this study, 13 ship models were built by 3D Max, and HRRP was calculated by FEKO. FEKO is 3D electromagnetic field simulation software, and is an abbreviation of “FEldberechnung für Körper mit beliebiger Oberfläche”, in German. When calculating the HRRP of a ship, the ship is stationary, and the HRRP of the ship in different directions is obtained by changing the incident direction of the electromagnetic wave. Since the ship is stationary when calculating HRRP, we do not apply three-dimensional rotation around the different Cartesian axes. The set simulation parameters include the center frequency of the radar as 10 GHz, the bandwidth as 80 MHz, the number of frequency sampling points as 256, the calculated azimuth range as 0–360°, and the interval as 1°. The grazing angle is 10°. The obtained HRRP has 256 range cells, with the corresponding length of each range cell as 1.875 m. The model and amplitude normalized HRRP of one of the ships are illustrated in Figure 5. Models of all of the ship targets are presented in Figure 6.

**Figure 7:**
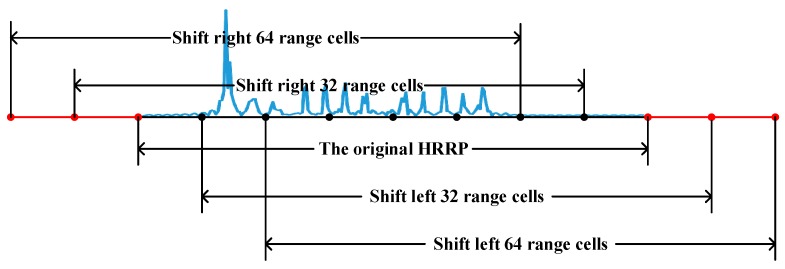
Interception of the HRRP.

In Figure 5b, the horizontal axis and the vertical axis represent HRRP length and azimuth angle, respectively. Each ship acquires 360 HRRP data. To meet the requirement of the data amount of the sample during neural network training and prevent over-fitting, the dataset should be expanded. The process is as follows:

1. Translation interception of HRRP. As revealed by Figure 5, when HRRP is calculated, the coordinate axis coincides with the center of the ship, so the effective HRRP information is generally in the middle region. However, when the radar detects the target, the echo signal may be incomplete or partially missing. Accordingly, the first step of data expansion is the translation interception of HRRP. Since each HRRP is one-dimensional data, only a one-dimensional translation interception is applied. The HRRP is shifted to the left and right by 32 and 64 range cells in turn. The data removed is discarded, and the blank part is supplemented with 0, as presented in Figure 7. The number of samples is increased to 5 times by taking those HRRP samples that overlap but are not identical. It should be noted that the translation interception of HRRP is to simulate the partially missing echo signal, and there is no spatial transformation performed on the object during the HRRP acquisition and expansion process.

2. Random noise is added to the translated HRRP data. Gaussian white noise was added to the data 10 times, and the data after adding noise meets a certain SNR.

2/3 of the target data of each class of ship are randomly taken as the training dataset and 1/3 as the testing dataset. In the database, the training dataset samples and the testing dataset samples were 156,000 and 78,000, respectively.

### 4.2. Model Identification Performance Analysis

In this section, the performance of the presented model is analyzed in three aspects. The first part primarily shows the effect of different loss functions on the recognition effect. The second part primarily analyzes the advantages of the presented model compared with the comparison model. The third part primarily analyzes the enhancement of model complexity to recognition effect.

The experiment was conducted under the following circumstances. Operating system: Windows 10. Memory: 64 GB. Video memory: 11 GB. GPU: NVIDIA GeForce RTX 2080 Ti. CPU: Intel(R) Xeon(R) w-2125 CPU @4.00GHz.

All the networks were trained from scratch. The iterations were set to 200. The learning rate began with 0.01, and it was halved every 20 training iterations. The Adam optimizer was employed to update the network weight. The batch gradient descent method was applied, and the number of training samples per batch was 512.

#### 4.2.1. Effect of Loss Function on Recognition Effect

The hyper-parameters of the presented model are limited to three types: the number of convolutional modules, the number of left branches in the modules and the parameters of the joint loss function.

To verify the effectiveness of the structure and loss function proposed, model A, with low complexity, is built first. The number of convolutional modules in model A is 4, and the number of left branches inside the module is 3. The parameters of the joint loss function are fine-tuned in accordance with the identification effect. Table 1 elucidates the structure and parameters of each stage in model A. After each convolutional layer, there are batch normalization and Relu activation operations. The number of parameters in the respective stages covers convolution kernel parameters and batch normalization parameters. For instance, the number of parameters of the initial convolutional layer is 63 + 36, suggesting 63 convolutional kernel parameters and 36 batch normalized parameters, respectively. The total number of parameters of model A is 37,538.

First, the effect of different loss functions on the recognition effect is compared under the structure of model A. The loss functions participating in the comparison refer to $L_{S}$, $L_{AMS}$ and $L_{SC}$.

##### Classification Effect Comparison of Loss Function $L_{AMS}$ and Loss Function $L_{S}$

The hyper-parameter μ in $L_{AMS}$ constrains the boundaries between features and s scales the cosine values. In [35], it was reported that the s will not increase, and the network converges in a relatively slow manner if the s is set to be learned. Thus, s is fixed at 30, which is a sufficiently large value. Thus, experiments are performed to delve into the sensitivity of parameter μ.

**Figure 8:**
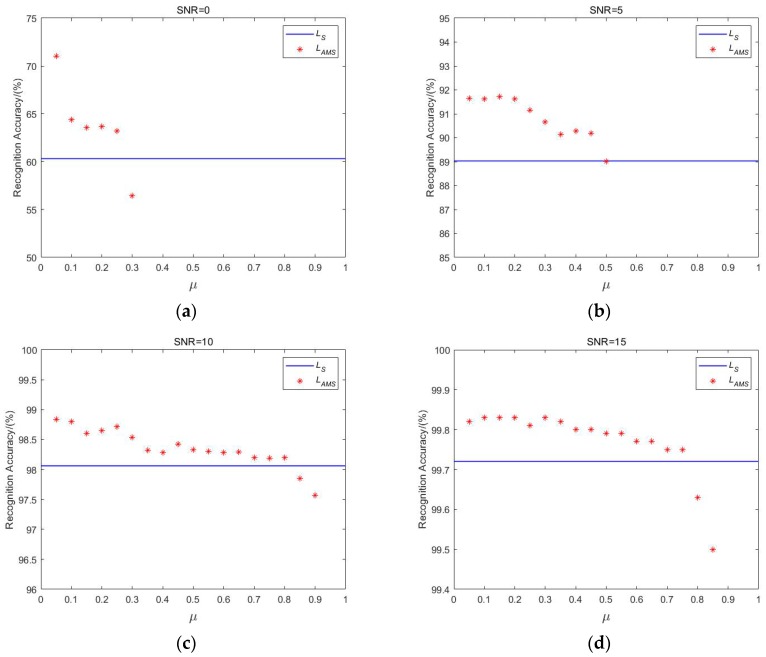
Recognition accuracy of model A with the dataset under different SNR conditions. (**a**) SNR = 0 dB; (**b**) SNR = 5 dB; (**c**) SNR = 10 dB; (**d**) SNR = 15 dB. The blue line suggests the recognition accuracy of model A using the loss function $L_{S}$, and the discrete red points indicate the recognition accuracy of model A using the loss function $L_{AMS}$ under different μ.

In the dataset with SNR of 0, 5, 10 and 15 dB, respectively. s is fixed to 30 and μ varies from 0 to 1 to compare the recognition accuracy of model A using loss function $L_{AMS}$ and $L_{S}$. The recognition accuracy is obtained by calculating the percentage of correctly classified samples in the testing dataset in the total number of samples, and the simulation results are presented in Figure 8. As suggested by Figure 8, compared with the conventional loss function $L_{S}$, the use of loss function $L_{AMS}$ improves the model recognition accuracy under different SNR conditions to a certain extent. In addition, the lower the SNR of the dataset is, the greater the enhancement in recognition accuracy. At different SNR, with the rise in the boundary constraint strength μ, the enhancement of recognition accuracy generally presents a downward trend. It is also noted that the effective range of boundary constraint strength is small when the SNR is low. In Figure 8a, the effective range of μ is only from 0 to 0.25 at SNR of 0 dB. Furthermore, the recognition accuracy after exceeding the range is lower than that with the use of loss function $L_{S}$ only. When the loss function $L_{AMS}$ is adopted for ship target recognition in our dataset, the value of boundary constraint strength should not be too large; 0.05 is generally appropriate, applying to a larger SNR range.

**Figure 9:**
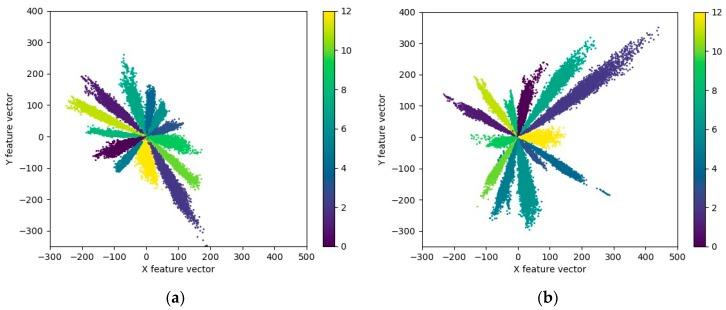
Visualization of the feature extracted by model A with a variety of loss functions at the SNR of 15 dB. (**a**) With the loss function $L_{S}$; (**b**) With the loss function $L_{AMS}$ and μ = 0.5. Each data point represents the two-dimensional feature extracted from HRRP data by the model A, and different colors represent different target types. The total number of types is 13.

To show the effect of loss function $L_{AMS}$ on the separability of features extracted from model A more intuitively, when the SNR is 15 dB, the testing dataset is visualized with the 2d features of the second full-connection layer in model A, as shown in Figure 9. It can be seen that after the loss function $L_{AMS}$ is used, the corner space occupied by the extracted features in sample of each class becomes smaller, the inter-class variations of features become larger, and the features are more separable. It is also noted that the scale of the feature increases with the use of the loss function $L_{AMS}$. That is, the features of the same class become more slender in terms of spatial distribution.

##### Classification Effect Comparison of Loss Function $L_{SC}$ and Loss Function $L_{S}$

When the weight λ is introduced to fuse the loss function $L_{S}$ with the loss function $L_{C}$, it yields $L_{SC}$. The hyper-parameter α in $L_{SC}$ is adopted to control the learning rate of center for the features, and λ is applied for the balance of the two functions. Experimental results reveal that when the learning rate α varies, the recognition accuracy fluctuates slightly. Here, to simplify model design and optimization, the learning rate α is directly fixed at 0.6. Therefore, we conduct experiments to investigate the sensitivity of parameter λ while the dataset under different SNR conditions. The simulation results are listed in Table 2.

**Figure 10:**
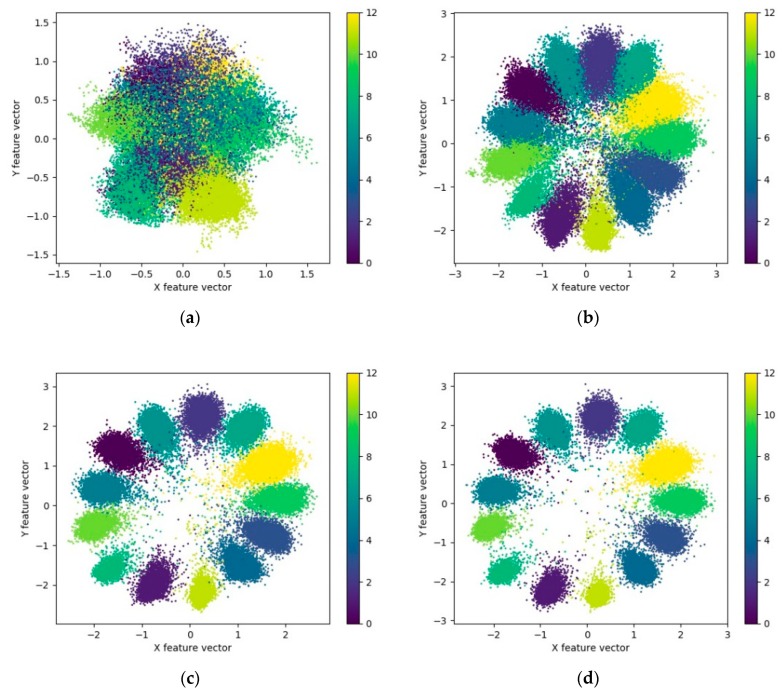

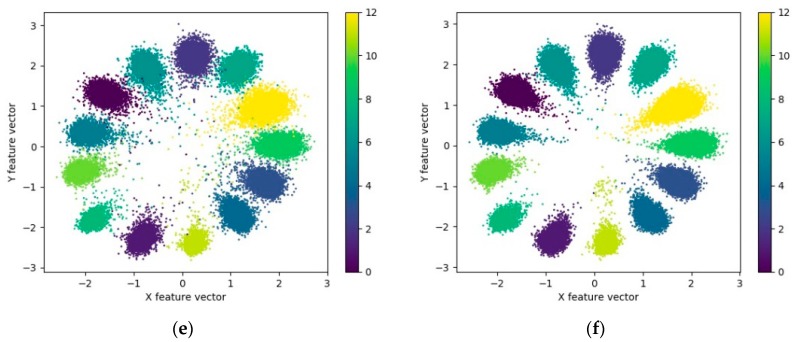
Visualization of the feature extracted by model A with the loss function $L_{SC}$ during training. (**a**–**e**) Feature visualization of testing dataset, and (**f**) feature visualization of training dataset. (**a**) Iterations = 1 and recognition accuracy = 0.3274; (**b**) Iterations = 50 and recognition accuracy = 0.9489; (**c**) Iterations = 100 and recognition accuracy = 0.9968; (**d**) Iterations = 150 and recognition accuracy = 0.9968; (**e**) Iterations = 200 and recognition accuracy = 0.9972; (**f**) Iterations = 200 and recognition accuracy = 0.9988.

Comparing the recognition accuracy in Table 2 with the results in Figure 8 using the loss function $L_{AMS}$, the loss function $L_{SC}$ is suggested to be more robust to noise, whereas it has a limited effect on the enhancement of recognition accuracy, indicating that reducing the intra-class variations of features alone cannot significantly enhance the recognition effect of the model. To show the process of establishing the center of features extracted by the loss function $L_{SC}$. When SNR of the dataset is 15 dB and the weight λ is 0.6, the 2d features of the second fully connected layer of the dataset in model A are visualized for every 50 iterations, as shown in Figure 10.

As suggested by Figure 10a, the initial features of each class are inseparable, and the initial recognition accuracy is only about 0.3274. With the increase in iteration times and the constant updating of parameters, the features of various samples are gradually separated and concentrated in their category centers. With the enhancement of feature separability, the model recognition accuracy rises. The comparison between (c) and (d) in Figure 10 suggests that though the recognition accuracy of the model is not improved between 100 and 150 iterations, the features of various samples are more clustered, and the intra-class variations of features are gradually decreased. As suggested by the comparison of Figure 10e,f, although the training dataset exhibits stronger feature separability and higher recognition accuracy, the testing dataset have similar feature distribution and recognition accuracy. It is, therefore, revealed that the model has no obvious overfitting and the extracted features have good generalization performance. Compared with the visualization of features extracted by model A when loss function $L_{AMS}$ and $L_{S}$ are used in Figure 9, the feature scale extracted by loss function $L_{SC}$ is smaller, and the distribution range is narrowed from [−400,400] to [−3,3]. The distribution of features in space varies from divergence to aggregation by class, and the intra-class difference is smaller.

##### Classification Effect Comparison between Loss Function $L_{SC}$ and Others

By analyzing the described results, it can be concluded that the boundary constraint strength μ of the loss function $L_{AMS}$ can significantly improve the recognition accuracy. However, when the SNR is low, the value of μ should not be overly large. The weight λ of loss function $L_{SC}$ has better adaptability and can improve the intra-class aggregation effect of features within a larger value range, but the enhancement of recognition accuracy is limited.

In this section, we verify the enhancement of recognition accuracy with the joint loss function $L_{AMSC}$, where s, α and μ are fixed at 30, 0.6 and 0.05, respectively. When λ is taken to have different values, the recognition accuracy of model A under different SNR conditions is listed in Table 3.

**Figure 11:**
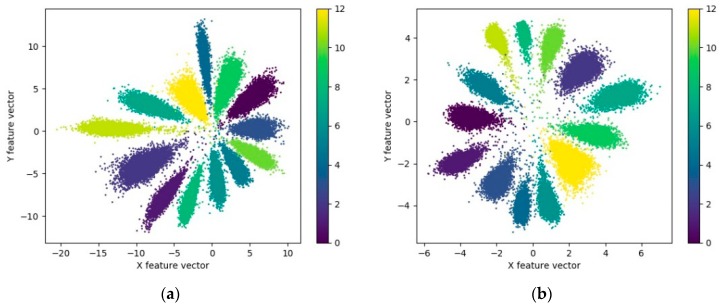

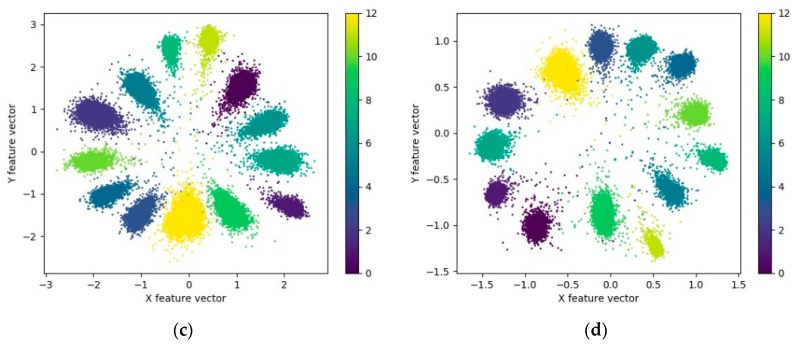
Visualization of the feature extracted by model A with different value of *λ*. (**a**) *λ* = 0.001; (**b**) *λ* = 0.01; (**c**) *λ* = 0.1; (**d**) *λ* = 1.

In Table 3, the recognition accuracy of model A is given when the λ in the joint loss function $L_{AMSC}$ is taken to have different values. In the meantime, it also shows the recognition accuracy when using loss function *L_AMS_*, *L_SC_* and *L_S_*. Among them, the loss function *L_AMS_* and *L_SC_* show the best recognition accuracy when assuming different values of parameters. As suggested by Table 3, when the joint loss function *L_AMSC_* is used, the recognition accuracy is improved stably under different SNR conditions. In addition, when the SNR of the dataset is relatively low, the enhancement is greater. When the value of *λ* is 0.001, 0.01, 0.1 and 1, respectively. we visualize the features extracted by model A, as shown in Figure 11.

As suggested by Figure 11, with increasing value of *λ*, the intra-class differences of features gradually become smaller, and the features of different types gradually converge to the center of the class. The spatial distribution range of features is narrowed from [−20, 15] to [−1.5, 1.5]. As suggested by the recognition accuracy and the intra-class aggregation of features, the recognition effect is identified the optimal at the value of *λ* as 0.1.

#### 4.2.2. Analysis of the Recognition Effect of the Presented Model and the Comparison Model

In this section, the common target recognition algorithm based on HRRP is selected as the comparison model to verify the effectiveness of the presented model and loss function. Conventional comparison algorithms based on machine learning include: KNN [36], LSVM [37], RBF-SVM [38], RF [39] and NB [40]. The comparison algorithms based on neural network includes: CNN [18], Stack Sparse Auto Encoder and K-Nearest Neighbor (sDSAE&KNN) [24], Stack Convolutional Auto Encoder (SCAE) [41]. For the highest recognition accuracy, the hyper-parameters in the comparison algorithm are fine-tuned. Table 4, Table 5 and Table 6 elucidate the structure and parameters of each comparison model based on the neural network. The pooling layer in each model is max-pooling, and batch normalization is performed after the convolutional layer in CNN.

Since the complexity of the model is associated with the recognition accuracy, the number of parameters of each model is similar to model A when the comparison model based on neural network is designed. The total parameters of the model are employed here to represent the complexity of the model. As suggested by the table above, the complexity of each model based on neural network is shown in descending sequence: sDSAE&KNN, SCAE, CNN, model A.

**Figure 12:**
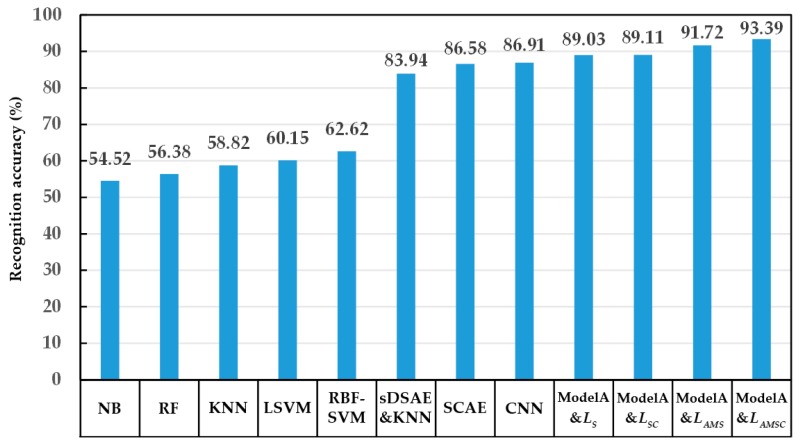
Recognition accuracy of different models when the dataset SNR is 5 dB.

First, the recognition effect of all models was compared using the dataset under the condition SNR = 5 dB. The recognition accuracy of each model is shown in Figure 12.

Figure 12 shows the best recognition accuracy of the comparison model and the model A with a variety of loss functions. As suggested in Figure 12, each model based on the proposed structure (model A) achieves better recognition effect. Additionally, the recognition of model A combined with the joint loss function *L_AMSC_* exhibits the highest accuracy among all models. The effectiveness of the proposed structure and the joint loss function is verified, respectively.

In the meantime, the recognition effect based on neural network model appears to be generally better than that based on conventional machine learning model. In the neural network models, the model including the convolution kernel (model A, CNN, SCAE) can achieve a prominent recognition effect. In the meantime, the model based on the convolutional neural network (model A, CNN) outperforms the model based on the auto-encoder (SCAE, sDSAE&KNN). During the expansion of the dataset, translation interception is performed to simulate target occlusion and information loss in the echo signal to some extent. The convolutional neural network-based recognition exhibits higher accuracy, revealing that the convolution kernel helps the model extract the effective separable features of different target echo signals, achieve better recognition effect, and avoid being adversely affected by incomplete echo signal information.

Under different SNR conditions, the optimal recognition results of each model based on neural network are listed in Table 7.

As suggested by the recognition results in Table 7, the recognition accuracy of each model noticeably impacts SNR. Additionally, the recognition accuracy of each model is enhanced with the rise in SNR of the dataset. Compared with the comparison model, model A exhibits the least number of parameters and the least complexity, whereas the highest recognition accuracy is achieved under different SNR datasets. By enhancing the network structure and loss function, the presented model achieves better recognition effect with less model complexity and exhibits higher generalization performance and noise robustness. It should be noted that the calculation process of the model proposed is more complicated, so it takes more time to identify the target.

#### 4.2.3. Effect of Model Complexity on Recognition Effect

The mentioned experimental results verify the effectiveness of the structure and loss function proposed. Since the recognition accuracy of the model displays a positive correlation with the depth and width of the model within a certain range. In the present section, different parameters will be selected, three models with different complexity will be designed, and their recognition effects will be compared. Model A refers to the model adopted in Section 4.2.1. Model B is developed by up-regulating the number of convolutional modules in model A to 5, and Model C is obtained by up-regulating the number of branches in the left branch of model A to 6. The details of the structure and parameters of each stage in model B and C are listed in Table 8 and Table 9.

Under different SNR conditions, the optimal recognition results of each model are listed in Table 10. The proposed joint loss function *L_AMSC_* is used in all models, and the values of each parameter of the loss function are *s* = 30, *α* = 0.6, *μ* = 0.05, *λ* = 0.1.

**Figure 13:**
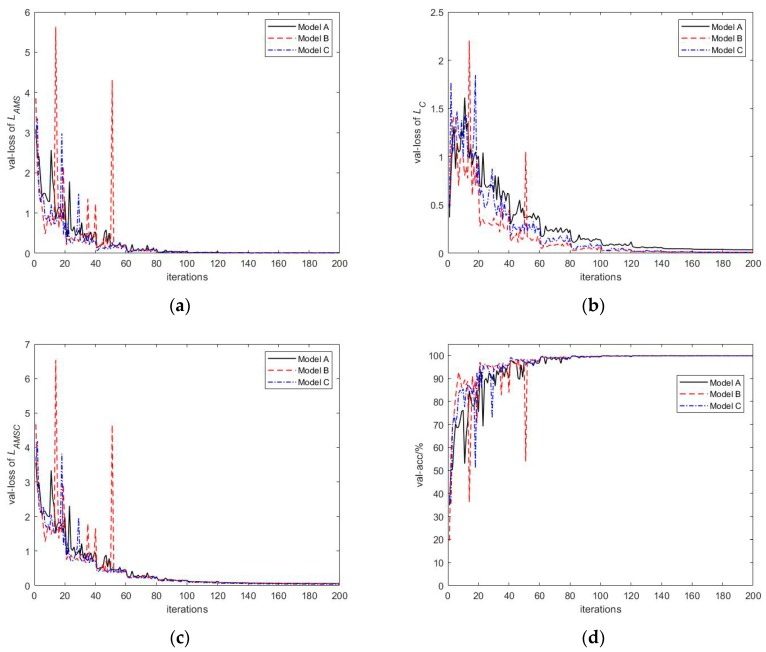
The recognition accuracy and loss curves in the training process at the dataset SNR of 15 dB, where val-loss and val-acc refer to the loss and recognition accuracy of the testing dataset, respectively. *L_AMS_* and *L_C_* refer to the combined parts of the joint loss function *L_AMSC_*, as shown in Equation (4). (**a**) The loss curve of *L_AMS_*; (**b**) The loss curve of *L_C_*; (**c**) The loss curve of *L_AMSC_*; (**d**) The accuracy curve.

As revealed by Table 10, the depth and width of the model directly impact the recognition effect. Compared with model A, models B and C both enhance the recognition accuracy noticeably. In particular, when the SNR is low, the enhancement becomes more obvious. Meanwhile, the time required for model A, B and C to calculate each HRRP is also listed in Table 10. It can be seen that compared with model A, the computational times for model B and model C increase due to the increased complexity of the model. However, compared with the increased number of parameters, the increase in calculation time is not large. To compare and delve into the convergence speed of various complexity models, at the dataset SNR of 15 dB, the recognition accuracy and loss curves in the training process are plotted in Figure 13.

**Figure 14:**
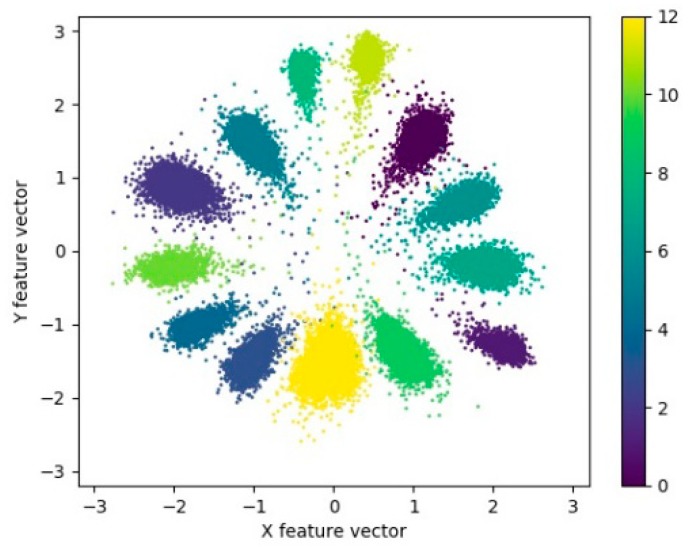
Visualization of the feature extracted by model A.

**Figure 15:**
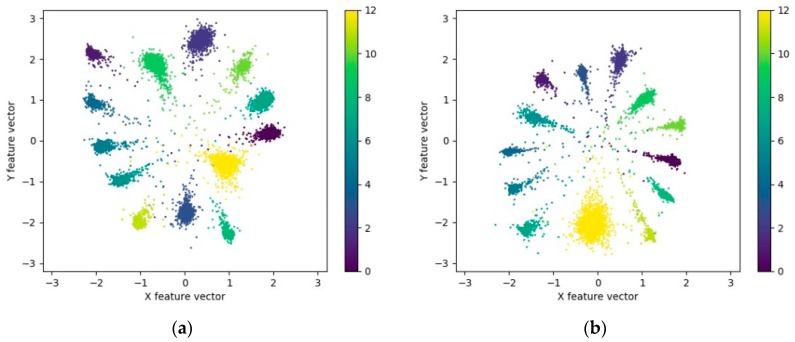
Visualization of the feature extracted by model B and C. (**a**) Model B; (**b**) Model C.

Figure 13 reveals that the recognition accuracy curve and the loss curve of model B and C fluctuate more dramatically during the training process, whereas they converge faster, as compared with those of model A. In the initial 60 iterations, the loss and the recognition accuracy curves of the testing dataset decline and increase rapidly. After 60 iterations, the model comes to exhibit a relatively high training effect. Subsequently, until the end of training, the loss and recognition accuracy gradually converge to stable values. In the meantime, the loss curve of model A in Figure 13b is always higher than that of models B and C, and a certain gap remains until the end of the training. The *L_C_* in the joint loss function *L_AMSC_* indicates the intra-class difference of the features extracted by the model, which suggests that the features extracted by model B and C undergo intra-class aggregation more effectively. The visualization of features also verifies this conclusion. The feature visualization of each model is illustrated in Figure 14 and Figure 15, respectively.

Though the model becomes more complex with the increases in depth and width, the presented model is capable of extracting deeper and more stable separable features in HRRP data for identification, thereby making the model more adaptable to SNR.

## 5. Conclusions

In this study, a neural network model integrating micro convolutional module and residual structure is proposed to classify ship targets based on HRRP. The model is characterized by few hyper-parameters, has easy to expand properties, and high recognition accuracy. The convolutional module is set as a simple and highly modular network structure that exhibits strong scalability. Based on the left branch structure of convolutional module, the effect of network deepening and widening can be simulated. The skip structure of the right branch is capable of transferring features and gradients more effectively. The presented model can up-regulate the utilization rate of shallow features while lowering the risk of gradient disappearance and recognition rate saturation. In the meantime, a novel loss function combining boundary constraint and center clustering is developed. The features extracted by the novel loss function are characterized by larger inter-class variations, smaller intra-class variations, as well as stronger separability. The effects of loss function and model complexity on recognition accuracy are analyzed by simulation experiments. Compared with other commonly used network structures, the presented model in this study exhibits higher recognition accuracy with fewer model parameters, good generalization performance and robustness.

## Figures and Tables

**Table 1:** Details of structure and parameters of each stage in model A.

Stage	Output Size	Structure			Number of Parameters
Initial convolutional layer	128 × 1 × 9	7 × 1, 9, s = 2			63 + 36
		Left branch		Right branch	
Convolutional module 1	64 × 1 × 18	1 × 1, 9 3 × 1, 3, s = 2, x = 3 1 × 1, 12		1 × 1, 15, s = 2	405 + 180
Convolutional module 2	32 × 1 × 36	1 × 1, 18 3 × 1, 6, s = 2, x = 3 1 × 1, 24		1 × 1, 30, s = 2	1620 + 360
Convolutional module 3	16 × 1 × 72	1 × 1, 36 3 × 1, 12, s = 2, x = 3 1 × 1, 48		1 × 1, 60, s = 2	6480 + 720
Convolutional module 4	8 × 1 × 144	1 × 1, 72 3 × 1, 24, s = 2, x = 3 1 × 1, 96		1 × 1, 120, s = 2	25,920 + 1440
Fully connected layer 1	144	Global max pooling and global average pooling			0
Fully connected layer 2	2				288
Output layer	13	Joint loss function			26
Total number of parameters			37,538		

**Table 2:** Recognition accuracy of model A when the λ in loss function $L_{SC}$ is different while the dataset is under different SNR conditions.

Loss Function and Parameter	Recognition Accuracy(%)			
	SNR = 0 dB	SNR = 5 dB	SNR = 10 dB	SNR = 15 dB
$L_{S}$	60.32	89.03	98.06	99.72
$L_{SC}$, λ = 0.001	60.45	89.10	98.07	99.73
$L_{SC}$, λ = 0.005	60.38	89.08	98.06	99.75
$L_{SC}$, λ = 0.01	60.42	89.08	98.07	99.75
$L_{SC}$, λ = 0.05	60.46	89.06	98.08	99.74
$L_{SC}$, λ = 0.1	60.40	89.11	98.09	99.73
$L_{SC}$, λ = 0.2	60.35	89.10	98.09	99.73
$L_{SC}$, λ = 0.4	60.37	89.08	98.07	99.74
$L_{SC}$, λ = 0.6	60.40	89.08	98.08	99.72
$L_{SC}$, λ = 0.8	60.38	89.09	98.07	99.74
$L_{SC}$, λ = 1	60.35	89.09	98.07	99.75

**Table 3:** Recognition accuracy of model A when the λ in loss function $L_{AMSC}$ is different while the dataset is under different SNR conditions.

Loss Function and Parameter	Recognition Accuracy(%)			
	SNR = 0 dB	SNR = 5 dB	SNR = 10 dB	SNR = 15 dB
$L_{AMSC}$, λ = 0.001, μ = 0.05	71.26	**93.39**	98.91	99.89
$L_{AMSC}$, λ = 0.01, μ = 0.05	70.58	93.16	99.03	99.89
$L_{AMSC}$, λ = 0.1, μ = 0.05	72.28	92.90	99.08	99.91
$L_{AMSC}$, λ = 0.2, μ = 0.05	69.70	92.46	98.89	99.91
$L_{AMSC}$, λ = 0.3, μ = 0.05	70.61	93.09	99.00	99.90
$L_{AMSC}$, λ = 0.4, μ = 0.05	71.14	92.36	98.99	99.90
$L_{AMSC}$, λ = 0.6, μ = 0.05	69.78	92.49	99.00	99.88
$L_{AMSC}$, λ = 0.8, μ = 0.05	70.92	92.85	99.08	99.91
$L_{AMSC}$, λ = 1, μ = 0.05	71.41	93.26	99.06	99.91
$L_{AMS}$	65.03	91.72	98.84	99.84
$L_{SC}$	60.46	89.11	98.09	99.75
$L_{S}$	60.32	89.03	98.06	99.72

**Table 4:** Details of structure and parameters of CNN.

Stage	Output Size	Structure	Number of Parameters
Convolutional layer 1	256 × 1 × 8	3 × 1, 8, s = 1	32 + 32
Pooling layer 1	128 × 1 × 8	2 × 1, s = 2	0
Convolutional layer 2	128 × 1 × 16	3 × 1, 16, s = 1	400 + 64
Pooling layer 2	64 × 1 × 16	2 × 1, s = 2	0
Convolutional layer 3	64 × 1 × 32	3 × 1, 32, s = 1	1568 + 128
Pooling layer 3	32 × 1 × 32	2 × 1, s = 2	0
Convolutional layer 4	32 × 1 × 64	3 × 1, 64, s = 1	6208 + 256
Pooling layer 4	16 × 1 × 64	2 × 1, s = 2	0
Convolutional layer 5	16 × 1 × 64	1 × 1, 64, s = 1	4160 + 256
Pooling layer 5	8 × 1 × 64	2 × 1, s = 2	0
Fully connected layer 1	64		32,832
Fully connected layer 2	2		130
Output layer	13	*L_s_*	39
Total number of parameters		46,105	

**Table 5:** Details of structure and parameters of sDSAE&KNN.

Stage	Output Size	Number of Parameters
Hidden layer 1	150 × 1	38,550
Hidden layer 2	100 × 1	15,100
Hidden layer 3	50 × 1	5050
Hidden layer 4	10 × 1	510
Total number of parameters	59,210	

**Table 6:** Details of structure and parameters of SCAE.

Stage	Output Size	Structure	Number of Parameters
Convolutional layer 1	256 × 1 × 128	5 × 1, 128, s = 1	768
Pooling layer 1	128 × 1 × 128	2 × 1, s = 2	0
Convolutional layer 2	128 × 1 × 64	5 × 1, 64, s = 1	41,024
Pooling layer 2	64 × 1 × 64	2 × 1, s = 2	0
Convolutional layer 3	64 × 1 × 32	3 × 1, 32, s = 1	6176
Pooling layer 3	32 × 1 × 32	2 × 1, s = 2	0
Convolutional layer 4	32 × 1 × 16	3 × 1, 16, s = 1	1552
Pooling layer 4	16 × 1 × 16	2 × 1, s = 2	0
Convolutional layer 5	16 × 1 × 8	1 × 1, 8, s = 1	136
Pooling layer 5	8 × 1 × 8	2 × 1, s = 2	0
Output layer	13	*L_s_*	845
Total number of parameters		50,501	

**Table 7:** Recognition accuracy of model A and the comparison model under different SNR conditions.

Model Name	Number of Parameters	Computational Time for Each HRRP (us)	Recognition Accuracy (%)			
			SNR = 0 dB	SNR = 5 dB	SNR = 10 dB	SNR = 15 dB
Model A&*L_AMSC_*	37538	258	72.28	93.39	99.08	99.91
CNN	46105	69	58.22	86.91	95.51	98.79
SCAE	50501	47	54.78	86.58	94.44	98.78
sDSAE&KNN	59210	68	46.50	83.94	93.44	98.65

**Table 8:** Details of structure and parameters of each stage in model B.

Stage	Output Size	Structure			Number of Parameters
Initial convolutional layer	128 × 1 × 9	7 × 1, 9, s = 2			63 + 36
		Left branch		Right branch	
Convolutional module 1	64 × 1 × 18	1 × 1, 9 3 × 1, 3, s = 2, x = 3 1 × 1, 12		1 × 1, 15, s = 2	405 + 180
Convolutional module 2	32 × 1 × 36	1 × 1, 18 3 × 1, 6, s = 2, x = 3 1 × 1, 24		1 × 1, 30, s = 2	1620 + 360
Convolutional module 3	16 × 1 × 72	1 × 1, 36 3 × 1, 12, s = 2, x = 3 1 × 1, 48		1 × 1, 60, s = 2	6480 + 720
Convolutional module 4	8 × 1 × 144	1 × 1, 72 3 × 1, 24, s = 2, x = 3 1 × 1, 96		1 × 1, 120, s = 2	25,920 + 1440
Convolutional module 5	4 × 1 × 288	1 × 1, 144 3 × 1, 48, s = 2, x = 3 1 × 1, 192		1 × 1, 240, s = 2	103,680 + 2880
Fully connected layer 1	144	Global max pooling and global average pooling			0
Fully connected layer 2	2				578
Output layer	13	Joint loss function			26
Total number of parameters			144,353		

**Table 9:** Details of structure and parameters of each stage in model C.

Stage	Output Size	Structure			Number of Parameters
Initial convolutional layer	128 × 1 × 18	7 × 1, 18, s = 2			126 + 72
		Left branch		Right branch	
Convolutional module 1	64 × 1 × 36	1 × 1, 18 3 × 1, 3, s = 2, x = 6 1 × 1, 24		1 × 1, 30, s = 2	1458 + 360
Convolutional module 2	32 × 1 × 72	1 × 1, 36 3 × 1, 6, s = 2, x = 6 1 × 1, 48		1 × 1, 60, s = 2	5832 + 720
Convolutional module 3	16 × 1 × 144	1 × 1, 72 3 × 1, 12, s = 2, x = 6 1 × 1, 96		1 × 1, 120, s = 2	23,328 + 1440
Convolutional module 4	8 × 1 × 288	1 × 1, 144 3 × 1, 24, s = 2, x = 6 1 × 1, 192		1 × 1, 240, s = 2	93,312 + 2880
Fully connected layer 1	288	Global max pooling and global average pooling			0
Fully connected layer 2	2				578
Output layer	13	Joint loss function			26
Total number of parameters			13,0132		

**Table 10:** Recognition accuracy of different complexity models under different SNR conditions.

Model Name	Number of Parameters	Computational Time for Each HRRP (us)	Recognition Accuracy (%)			
			SNR = 0 dB	SNR = 5 dB	SNR = 10 dB	SNR = 15 dB
Model A	37538	258	72.28	92.90	99.08	99.91
Model B	144353	326	77.12	95.28	99.49	99.93
Model C	130132	323	76.31	95.50	99.43	99.93
